# High prevalence of multidrug resistance in bacterial uropathogens from Kathmandu, Nepal

**DOI:** 10.1186/1756-0500-5-38

**Published:** 2012-01-19

**Authors:** Pankaj Baral, Sanjiv Neupane, Bishnu Prasad Marasini, Kashi Ram Ghimire, Binod Lekhak, Basudha Shrestha

**Affiliations:** 1 Central Department of Microbiology, Tribhuvan University, Kirtipur, Kathmandu, Nepal; 2 Department of Microbiology, Faculty of Science, Mahidol University, Bangkok, Thailand; 3 Kathmandu Model Hospital, Kathmandu, Nepal

**Keywords:** Urinary tract infection, Multidrug resistance, Plasmid

## Abstract

**Background:**

Urinary Tract Infection (UTI) is one of the most common infectious diseases and people of all age-groups and geographical locations are affected. The impact of disease is even worst in low-resource developing countries due to unaware of the UTIs caused by multidrug-resistant (MDR) pathogens and the possibility of transfer of MDR traits between them. The present study aimed to determine the prevalence of MDR bacterial isolates from UTI patients, the antibiotic resistance pattern and the conjugational transfer of multidrug resistance phenotypes in *Escherichia coli (E. coli*).

**Results:**

Two hundred and nineteen bacterial isolates were recovered from 710 urine samples at Kathmandu Model hospital during the study period. All samples and isolates were investigated by standard laboratory procedures. Among the significant bacterial growth (30.8%, 219 isolates), 41.1% isolates were MDR. The most prevailing organism, *E. coli *(81.3%, 178 isolates) was 38.2% MDR, whereas second most common organism, *Citrobacter *spp. (5%, 11 isolates) was found 72.7% MDR. Extended-spectrum β-lactamase (ESBL) production was detected in 55.2% of a subset of MDR *E. coli *isolates. Among the 29 MDR *E. coli *isolates, plasmids of size ranging 2-51 kb were obtained with different 15 profiles. The most common plasmid of size 32 kb was detected in all of the plasmid-harbored *E. coli *strains. The majority of *E. coli *isolates investigated for the multidrug resistance transfer were able to transfer plasmid-mediated MDR phenotypes along with ESBL pattern with a frequency ranging from 0.3 × 10^-7 ^to 1.5 × 10^-7 ^to an *E. coli *HB101 recipient strain by conjugation. Most of the donor and recipient strain showed high levels of minimum inhibitory concentration (MIC_) _values for commonly-used antibiotics.

**Conclusions:**

The high prevalence of multidrug resistance in bacterial uropathogens was observed. Particularly, resistance patterns were alarmingly higher for amoxycillin, co-trimoxazole, flouroquinolones and third-generation cephalosporins, which necessitate the re-evaluation of first and second line therapies for UTI. In addition, conjugational co-transfer of MDR phenotypes with ESBL-positive phenotypes was observed in MDR *E. coli*.

## Background

Urinary tract infection is a common bacterial disease, often contributes to a frequent cause of morbidity in out-patients as well as hospitalized-patients [1]. Clinical experience has indicated the presence of numerous cases of antibiotic resistance to common antibiotics by uropathogens in both developed and developing countries [2]. Resistant to newer and more potent antimicrobials are no exceptions, making the therapeutic options very limited to certain antimicrobial agents like carbapenem, colistin and fosfomycin [3]. In many parts of Nepal, the facilities for urine culture and antimicrobial susceptibility testing are still not available, leading to improper diagnosis and irrational antibiotic treatment (e.g. self-medication) of UTI [4]. The updated knowledge and situation of the prevailing bacterial uropathogens that are multidrug resistant (MDR) is of prime importance for the proper use of antimicrobial drugs and the policy making to combat multidrug resistance in UTIs [3].

Invariably, *Escherichia coli *(*E. coli) *has been found as a most common uropathogen in a number of reports worldwide [5-7]. Antimicrobial therapy of UTI caused by *E. coli *is often impaired due to the resistance to commonly-used antimicrobial agents [3,5]. Although *E. coli *has been reported to be MDR by possessing the antibiotic resistant genes in its transferable R-plasmid (s) [8], detection of this feature in UTI isolates from Nepal is largely unknown. The present study aimed to determine the prevalence of MDR bacterial isolates in UTI, the antibiotic resistance pattern, the plasmid profile of isolated MDR *E. coli*, and the transfer of multidrug resistance phenotypes by conjugation. Our data might be informative to both of the health professionals and the scientific community, which may help to make a positive contribution to current understanding and knowledge of the situation in UTI caused by MDR bacterial pathogens.

## Methods

### Study population and bacterial isolates

This prospective study was carried out at Kathmandu Model Hospital between 1st May and 30th September 2007. This public hospital is one of big hospitals located in capital, Kathmandu, where patients across the country visit regularly. Conduction of this study was approved by ethics and research committee of Kathmandu Model Hospital. Written informed consents were obtained from all cases prior to their inclusion in the study. All suspected patients of UTI during the study period were interviewed directly with structured questionnaire to collect data about patient's demographics, type of patient (hospitalized- or out-patient), symptoms, prior history of UTI, and underlying diseases. Only those cases with at least one of the clinical features of UTI (dysuria, frequency, urgency, loin pain or prior history of UTI) were judged as clinically-suspected patients of UTI and selected for confirmation of UTI. A total of 710 mid-stream 'clean-catched' urine samples were collected from the same number of clinically-suspected patients of UTI. Once the sample was collected, transferred to the laboratory immediately and inoculated on blood agar and McConkey agar using a standard calibrated loop. Isolates from cases with significant bacteriuria (10^5 ^colonies/ml) were identified based upon standard laboratory procedures involving, morphological characteristics, Gram's stain, rapid tests (catalase, oxidase, coagulase, bile solubility), and biochemical tests (IMViC [Indole, methyl red, Voges-Proskauer and citrate], TSI [triple sugar iron], O/F [oxidation/fermentation], urease and nitrate reduction) [9]. Single isolate was selected from each of diagnosed samples. UTI diagnosis was established on the basis of pyuria (≥5 leukocytes per high-power field on microscopic examination of the urine), clinical feature (s) (as mentioned above) and significant bacteriuria.

### Antimicrobial susceptibility testing and MIC testing

Antimicrobial susceptibility patterns were determined according to the Clinical and Laboratory Standards Institute (CLSI)-recommended modified Kirby-Bauer disc diffusion method on Mueller-Hinton agar with commercial antibiotic discs (Oxoid Ltd, UK) [10]. Susceptibility to eight antimicrobial agents (amoxycillin, cefotaxime, cefixime, ciprofloxacin, cotrimoxazole, nitrofurantoin, norfloxacin and ofloxacin; used as primary) was determined for 209 g- negative bacterial isolates. Of these, 56 isolates were tested by additional antibiotic discs (amikacin, chloramphenicol, ceftazidime, ceftriaxone and gentamycin; used as secondary). Susceptibility to seven antimicrobial agents (amoxycillin, cephalexin, ciprofloxacin, cotrimoxazole, cloxacillin, erythromycin and norfloxacin) was determined for all 10 g- positive isolates. Four of these isolates were also tested by an additional antibiotic disc (vancomycin). Antibiotic concentrations in the diffusion discs used for antimicrobial susceptibility testing are: amikacin (30 μg), amoxycillin (10 μg), cefixime (5 μg), cefotaxime (30 μg), ceftazidime (30 μg), ceftriaxone (30 μg), cephalexin (30 μg), chloramphenicol (30 μg), ciprofloxacin (5 μg), cloxacillin (5 μg), co-trimoxazole (1.25/23.75 μg), erythromycin (15 μg), gentamycin (10 μg), nitrofurantoin (300 μg), norfloxacin (10 μg), ofloxacin (5 μg) and vancomycin (30 μg). *Escherichia coli *(ATCC 25922) and *Staphylococcus aureus *(ATCC 25923) were used as controls for Gram-negative and Gram positive bacteria, respectively.

Those organisms which showed resistance to at least three or more antibiotics of different structural classes were considered MDR as described elsewhere [11,12]. MDR *E. coli *isolates were preserved in tyrptic soy broth containing 20% glycerol at -70°C until further investigation.

Minimum inhibitory concentration (MIC) for donors and transconjugants was determined by two fold broth macrodilution method as recommended by CLSI [13]. The inocula of 4 × 10^5 ^colony forming unit (CFU) were prepared from suitably diluted overnight broth culture at 37°C. Positive growth controls were kept for each isolates and *E. coli *25922 of known MIC was also included in each test as control for antibiotic potency. MIC was defined as the minimum concentration of antimicrobial agent that prevented the visible growth of bacteria. The range of antibiotic concentrations employed in MIC test is: ampicillin (1-512 μg/ml), ciprofloxacin (0.016-256 μg/ml), cefixime (1-512 μg/ml), chloramphenicol (1-512 μg/ml), norfloxacin (0.016-256 μg/ml), co-trimoxazole (1-512 μg/ml) and gentamycin (0.5-64 μg/ml). The antibiotic powders were provided by National Drug Laboratory, Kathmandu, Nepal

### Determination of ESBL production

The ESBL-producing phenotypes from the 29 MDR *E. coli *isolates were determined by Kirby-Bauer disc diffusion method following CLSI recommendation. Amoxy-clavulanic acid (20/10 μg) disc (Oxoid Ltd, UK) was placed in the center of ceftazidime (30 μg) and cefotaxime (30 μg) discs with 25-30 mm apart. After overnight incubation at 37°C in air, confirmation of ESBL-producing organism was assessed when the zone of inhibition around ceftazidime and cefotaxime was expanded by at least 5 mm by the presence of clavulanic acid [14]. *E. coli *ATCC 25922 and *Klebsiella pneumoniae *ATCC 700603 were used as negative and positive controls for ESBL production, respectively.

### Plasmid profiles and conjugation

Plasmid DNA from MDR *E. coli *was extracted from the overnight culture (mid log phase) grown in Lauria-Bertani (LB) broth supplemented with antibiotic (100 μg/ml of ampicillin). The plasmid DNA was extracted and purified using a QIAprep spin miniprep kit (Qiagen, Germany) according to the manufacturer's instruction. Considering that the isolation of large plasmid may be affected using miniprep, isolation was carried out additional two times for those large plasmid ( > 20 kb) to be ensured about the proper plasmid size and number in the isolates. The plasmid DNA was analyzed through 0.5% horizontal agarose gel electrophoresis. Electrophoresis was run at 0.6 Vcm^-2 ^for 4 h in TAE buffer (40 mM Tris-acetate, 1 mM EDTA). Reference supercoiled plasmid DNA marker (New England Biolab, England) and plasmid from *E. coli *V517 were used for the estimation of plasmid sizes.

The MDR *E. coli *strains showing sensitivity to streptomycin were selected for conjugation study, serving as donors. The plasmid-free, streptomycin-resistant strain of *E. coli *(*E. coli *HB 101), showing sensitivity to all antibiotics under study, was used as a recipient. Conjugation was carried out as similarly described elsewhere [8]. Briefly, all donor *E. coli *strains and recipient *E. coli *strain were grown with antibiotic supplement till the O.D. of each type reaches 0.85 at A_600 _(approximately 10^8 ^cells/ml). Each of 0.2 ml donor and recipient culture broth were mixed together in 2.5 ml warm LB broth and incubated at 37°C without shaking. After 24 h. of incubation, bacteria were then plated onto selective LB agar supplemented with streptomycin (100 μg/ml) and at least one of the following antibiotics: ampicillin (30 μg/ml), ciprofloxacin (0.045 μg/ml), cefixime (2 μg/ml), chloramphenicol (20 μg/ml), norfloxacin (0.3 μg/ml), trimethoprim (200 μg/ml), cefotaxime (2 μg/ml) and gentamycin (10 μg/ml). It should be noted that the indicated concentrations of above antibiotics inhibited the growth of recipient strain *E. coli *HB101 only, but not donors. The transconjugants were identified based upon the visible growth on these counter-antibiotic containing medium and subjected for plasmid profiling. To ensure the transconjugants selection only by conjugation, not by mutation that can alter the streptomycin susceptibility in the donor strains, controls (for donor and recipient strain) were run in each conjugation experiment parallely, involving similar procedure of conjugation, but without adding recipient culture broth (for donor control) and donor culture broth (for recipient control). Successful conjugation was ascertained by finding no growth from controls, while visible growth from the conjugation mixture bacteria, in LB agar supplemented with streptomycin and at least one of aforementioned antibiotics. The antibiogram of the donors and transconjugants were studied by disc diffusion method and MIC testing as described above.

### Statistical analysis

The data was analyzed by using Minitab version 15 (Minitab Inc., USA). Statistical tests of significance were performed using the Kruskall-Wallis test. A *p *value of *≤ *0.05 was considered significant.

## Results

### Bacterial uropathogens

Two-hundred and nineteen bacterial pathogens (30.8%) were isolated from the 710 clinically-suspected cases of UTIs. High incidences of UTI cases were observed, among female patients (33.5%, 173/516) than male patients (23.7%, 46/194) (*p *< 0.05), and in hospitalized-patients (51.3%, 20/39) than out-patients (29.7%, 199/671) (*p *< 0.05). Out of the 219 isolates, Gram-negative bacilli accounted for 95.4% while Gram-positive cocci accounted for the remaining 4.6% of the total isolates. The most common urinary pathogen isolated was *E. coIi *(81.3%), followed by *Citrobacter *spp. (5%) and coagulase-negative staphylococci (CoNS) [2.7%; *S. epidermidis *(1.8%) and *S. saprophyticus *(0.9%)] (Table 1). The percent resistance to antibiotics shown by the Gram-negative pathogens are shown in Table 2. *E. coli *showed the high resistance rate against most of the primary drugs tested: amoxycillin (55.6%), co-trimoxazole (54.4%), norfloxacin (36.5%), ciprofloxacin (35.9%) and cefixime (30.3%). Comparatively, it showed low resistance rate against a primary drug, nitrofurantoin (7.9%). Out of the 13 antibiotics tested for *E. coli *isolates, the least resistance was observed to amikacin (6.2%) (Table 2).

**Table 1:** Distribution of MDR bacterial uropathogens

Organism	In hospitalized-patient		In out-patient		Total	
	
	No of isolates (%)	No of MDR isolates (%)	No of isolates (%)	No of MDR isolates (%)	No of isolates (% of total)	No of MDR isolates (%)
*Acinetobacter *spp.	1	1 (100)	1	0(0)	2 (0.9)	1 (50)
*Citrobacter *spp.	5	5 (100)	6	3 (50)	11 (5)	8 (72.7)
*Enterobacter *spp.	0	0 (0)	4	1 (25)	4 (1.8)	1 (25)
*Escherichia coli*	11	8 (72.7)	167	60 (35.9)	178 (81.3)	68 (38.2)
*Klebsiella oxytoca*	0	0 (0)	2	1 (50)	2 (0.9)	1 (50)
*K. pneumoniae*	0	0 (0)	4	1 (25)	4 (1.8)	1 (25)
*Morganella morganii*	0	0 (0)	2	0 (0)	2 (0.9)	0 (0)
*Proteus mirabilis*	0	0 (0)	3	0 (0)	3 (1.4)	0 (0)
*Pseudomonas aeruginosa*	0	0 (0)	2	2 (100)	2 (0.9)	2 (100)
*Salmonella typhi*	1	1(100)	0	0(0)	1 (0.5)	1 (100)
*Staphylococcus aureus*	0	0(0)	1	1 (100)	1 (0.5)	1 (100)
CoNS*	1	1 (100)	5	2 (40)	6 (2.7)	3 (50)
*Enterococcus faecalis*	1	1(100)	2	2 (100)	3 (1.4)	3 (100)
Total	20	17 (85)	199	73 (36.7)	219 (100)	90 (41.1)

*CoNS = coagulase-negative staphylococci

**Table 2:** Number (percent) of Gram-negative urinary pathogens resistant to antimicrobial agents

Antibiotic	*Escherichia coli*	***Klebsiella *spp**.	*Proteus mirabilis*	*Pseudomonas aeruginosa*	***Enterobacter *spp**.	***Citrobacter *spp**.	***Acinetobacter *spp**.	*Salmonella typhi*	*Morganella morganii*	No of resistant isolates/total isolates (% resistance)	
Amoxycillin(Am)	99/178(55.6)	6/6(100)	1/3(33.3)	NT	NT	8/11(72.7)	1/2(50)	1/1(100)	NT	116/201	**(57.7)**
Cefotaxime(Ce)	54/178(30.3)	2/6(33.3)	0/3(0)	1/2(50)	1/4(25)	7/11(63.6)	1/2(50)	0/1(0)	0/2(0)	66/209	**(31.6)**
Ciprofloxacin(Cf)	64/178(35.9)	1/6(16.7)	0/3(0)	1/2(50)	0/4(0)	6/11(54.5)	0/2(0)	1/1(100)	0/2(0)	73/209	**(34.9)**
Cefixime(Cfx)	54/178(30.3)	2/6(33.3)	0/3(0)	2/2(100)	1/4(25)	8/11(72.7)	1/2(50)	0/1(0)	0/2(0)	68/209	**(32.5)**
Cotrimoxazole(Co)	97/178(54.4)	2/6(33.3)	1/3(33.4)	2/2(100)	1/4(25)	8/11(72.7)	1/2(50)	0/1(0)	0/2(0)	112/209	**(53.6)**
Nitrofurantoin(Nf)	14/178(7.9)	4/6(66.6)	NT	NT	1/4(25)	5/11(45.4)	2/2(100)	1/1(100)	NT	27/202	**(13.4)**
Norfloxacin(Nx)	65/178(36.5)	1/6(16.7)	0/3(0)	2/2(100)	0/4(0)	6/11(54.5)	1/2(50)	1/1(100)	0/2(0)	76/209	**(36.4)**
Ofloxacin(Of)	63/178(35.4)	1/6(16.7)	0/3(0)	2/2(100)	0/4(0)	4/11(36.4)	1/2(50)	1/1(100)	0/2(0)	72/209	**(34.4)**
Amikacin(Ak)	3/48(6.2)	0/1(0)	NT	0/2(0)	NT	4/5(80)	0/2(0)	0/1(0)	NT	7/59	**(11.9)**
Chloramphenicol(C)	13/48(27.1)	1/1(100)	NT	2/2(100)	NT	5/5(100)	0/2(0)	0/1(0)	NT	21/59	**(35.6)**
Ceftazidime(Ca)	48/48(100)	1/1(100)	NT	2/2(100)	NT	5/5(100)	1/2(50)	0/1(0)	NT	57/59	**(96.6)**
Ceftriaxone(Ci)	48/48(100)	0/1(0)	NT	2/2(100)	NT	3/5(60)	1/2(50)	0/1(0)	NT	54/59	**(91.5)**
Gentamycin(G)	35/48(72.9)	1/1(100)	NT	2/2(100)	NT	5/5(100)	0/2(0)	0/1(0)	NT	43/59	**(72.9)**

The numbers within non-parentheses indicate the number of resistant isolates by total number isolates and the numbers within parentheses indicate the percent resistance

*NT*: not tested

The most predominant Gram-positive isolates, CoNS, showed a least resistance (16.7% in each) against the primary antibiotics (amoxycillin, ciprofloxacin, erythromycin and norfloxacin).

### Multidrug resistance in bacterial uropathogens

Out of the 219 isolates, 41.1% were MDR according to the mentioned criteria. High incidence of UTIs caused by MDR pathogens was observed, among male patients (58.7%, 27/46) than female patients (36.42%, 63/173) (*p *< 0.05), also in hospitalized-patients (85%, 17/20) than out-patients (36.7%, 73/199) (*p *< 0.05). Although multidrug resistance was shown 100% by *Enterococcus faecalis, Salmonella typhi, Staphylococcus aureus *and *Pseudomonas aeruginosa*, these were low in number and considered insignificant. Out of the 178 *E. coli *isolates, 38.2% were confirmed to be MDR (Table 1). *E. coli *(75.5%, 68/90) and *Citrobacter *spp. (8.9%, 8/90) shared the major part of MDR isolates (Table 1). The MDR *E. coli *isolates demonstrated the extended resistance pattern to different classes of antibiotics, such as 7.3% isolates showed resistance to 5-6 classes (Table 3).

**Table 3:** Distribution of MDR isolates resistant to indicated class range of antibiotics

Organism	3-4 classes		5-6 classes		≥7 classes		Total	
	**n**	**%**	**n**	**%**	**n**	**%**	**n**	**%**
*Acinetobacter *spp.(n = 2)	1	50	0	0	0	0	1	50
*Citrobacter *spp. (n = 11)	6	54.5	1	9.1	1	9.1	8	72.7
CoNS* (n = 6)	1	16.7	2	33.3	0	0	3	50
*E. coli *(n = 178)	54	30.3	13	7.3	1	0.6	68	38.2
*Enterobacter *spp.(n = 4)	1	25	0	0	0	0	1	25
*K. oxytoca *(n = 2)	1	50	0	0	0	0	1	50
*K. pneumoniae *(n = 4)	1	25	0	0	0	0	1	25
*M. morganii *(n = 2)	0	0	0	0	0	0	0	0
*P. mirabilis *(n = 3)	0	0	0	0	0	0	0	0
*P. aeruginosa *(n = 2)	0	0	2	100	0	0	2	100
*S. typhi *(n = 1)	1	100			0	0	1	100
*S. aureus *(n = 1*)*	0	0	0	0	1	100	1	100
*E. faecalis *(n = 3)	2	66.7	1	33.3	0	0	3	100
Total (n = 219)	68	31	19	8.7	3	1.4	90	41.1

*CoNS = coagulase-negative staphylococci

Majority of MDR *E. coli *isolates showed high resistance to different classes of antibiotics used: co-trimoxazole (86.8%), penicillin (amoxycillin, 94.1%), quinolone (ciprofloxacin and ofloxacin [92.6% in each]), cephalosporin (cefotaxime [79.4%] and cefixime [77.9%]) and nitrofurantoin (17.6%). Similarly, these MDR isolates were also shown high antibiotic resistance among the secondary antibiotics evaluated e.g. cephalosporin (ceftazidime and ceftriaxone [100% in each]), aminoglycosides (gentamycin [72.9%] and amikacin [6.2%]) and chloramphenicol (27.1%). The most common three types of multidrug resistance pattern by these MDR *E. coli *isolates, in respective order, include resistance to: amoxycilin, ciprofloxacin, cephotaxime, cefixime, cotrimoxazole, ofloxacin, norfloxacin, ceftazidime, gentamycin, ceftriaxone; amoxycilin, ciprofloxacin, cephotaxime, cefixime, cotrimoxazole, ofloxacin, norfloxacin, ceftazidime and amoxycilin, ciprofloxacin, cefixime, cotrimoxazole, ofloxacin, norfloxacin, ceftazidime, chloramphenicol, gentamycin, ceftriaxone.

### ESBL production, plasmid profiling and multidrug resistance transfer

Out of the 68 MDR *E. coli *isolates, 29 isolates representing the most common MDR patterns were further analyzed for ESBL production and plasmid profiling. ESBL production was detected in 16 (55.2%) isolates. All MDR *E. coli *isolates that were analyzed for plasmid profiles harbored at least single plasmid. Altogether 15 different types of plasmid profiles of size ranging from 2 to 51 kb were detected. Of which, 12 isolates showed single plasmid, 3 showed double plasmids, whereas 6, 6, 1 and 1 isolates showed 3, 4, 5, and 7 plasmids, respectively. The most common plasmid of size 32.5 kb was detected in the majority of the plasmid-harbored MDR *E. coli *strains. Out of the 29 plasmid-harbored MDR *E. coli *isolates, 10 isolates were screened and selected for conjugation study. Each of the isolates containing either single or multiple plasmids can transfer single or multiple plasmids to the recipient strain by conjugation. The highest transfer of plasmid of size 51 kb was detected by conjugation experiment. The MDR pattern (amoxycillin-, ciprofloxacin-, cefixime-, cotrimoxazole-, ofloxacin-, and norfloxacin-resistance) was found most likely to be transferred to recipient strain by conjugation with highest conjugation frequency of 1.5 × 10^-7^(Table 4).

**Table 4:** Conjugational transfer of multidrug resistance phenotypes in E. *coli*

Donor	Transconjugant						^c^MIC (μg/ml) of antibiotics towards donors and transconjugants							^a^Conjugation frequency
**Resistance pattern**	**Plasmid (kb)**	**^b^ESBL activity**	**Resistance pattern**	**Plasmid (kb)**	**^b^ESBL activity**		**Am**	**C**	**Cf**	**Cfx**	**G**	**Nx**	**Co**	
Am, Cf, Ce, Cfx, Co, Of, Nx, Ca, C	32.5	-	Am, Cf, Cfx, Co, Of, Nx	32.5	-	D	> 512		> 256	> 512		128	512	0.3 × 10^-7^
						T	> 512		64	> 512		64	512	
Am, Cf, Ce, Cfx, Co, Of, Nx, Ca, C, G, Ci	32.5	+	Am, Cf, Cfx, Co, Of, Nx	32.5	+	D	> 512		> 256	> 512		> 256	> 512	0.6 × 10^-7^
						T	> 512		64	512		64	> 512	
Am, Cf, Co, Nx, Na, Of, Ca, Ci	32.5	+	Am, Cf, Cfx, Co, Of, Nx	32.5	+	D	> 512		> 256	> 512		> 256	> 512	1.5 × 10^-7^
						T	> 512		128	> 512		64	512	
Am, Cf, Ce, Cfx, Co, Of, Nx, Ca, G, Ci	32.5	-	Am, Cf, Cfx, Co, Of, Nx	32.5	-	D	512		> 256	> 512		> 256	> 512	0.6 × 10^-7^
						T	512		64	512		64	512	
Am, Cf, Ce, Cfx, Co, Of, Nx, Ca, C, G, Ci	32.5, 4.7	+	Am, Cf, Cfx, Co, Of, Nx, C, G	32.5	+	D	> 512	512	> 256	> 512	> 64	> 256	> 512	1.2 × 10^-7^
						T	> 512	512	128	512	> 64	128	> 512	
Am, Cf, Ce, Cfx, Co, Of, Nx, Ca, G, Ci	51	+	Am, Cf, Cfx, Co, Of, Nx	51	-	D	512		> 128	512		128	512	0.5 × 10^-7^
						T	256		32	256		32	512	
Am, Cf, Ce, Cfx, Co, Of, Nx, Ak, Ca, G, Ci	38	+	Am, Cf, Cfx, Co, Of, Nx	38	+	D	> 512		> 256	> 512		> 256	> 512	0.8 × 10^-7^
						T	> 512		64	> 512		64	> 512	
Am, Cf, Co, Nx, Ca, Ci	38	-	Am, Cf, Cfx, Co, Of, Nx	38	-	D	512		128	> 512		128	512	0.5 × 10^-7^
						T	256		64	256		64	512	
Am, Cf, Ce, Cfx, Co, Of, Nx, C, Ca, G, Ci	32.5, 6.5	+	Am, Cf, Cfx, Co, Of, Nx	32.5, 6.5	+	D	512		128	> 512		128	> 512	0.3 × 10^-7^
						T	512		64	> 512		64	> 512	
Am, Cf, Ce, Cfx, Co, Of, Nx, Ca, Ci	32.5	+	Am, Cf, Cfx, Co, Of, Nx	32.5	+	D	> 512		128	> 512		128	> 512	1.2 × 10^-7^
						T	> 512		64	> 512		64	512	

^a^Conjugation frequency is the number of transconjugants per donor cell

^b^*ESBL *= extended-spectrum β-lactamase

^c^*MIC *= minimum inhibitory concentration of antibiotic at which visible growth of isolates were inhibited

Am-amoxycillin, Ce-cefotaxime, Cfx-cefixime, Cf-ciprofloxacin, Co-cotrimoxazole, Nf-nitrofurantoin, Nx-norfloxacin, Of-ofloxacin, Ak-amikacin, C-chloramphenicol, Ca-ceftazidime, Ci-ceftriaxone, G-gentamycin, D-donor, T-transconjugant

Majority of donor strains (85.7%, 6 isolates) can transfer the ESBL production phenotype to the recipient strain by conjugation (Table 4). Most of the donors and transconjugants showed high MIC values for the antibiotics tested, upto ≥512 μg/ml (ampicillin, chloramphenicol, cefixime, and trimethoprim) and ≥64 μg/ml (ciprofloxacin, norfloxacin and gentamycin) (Table 4).

## Discussion

The present study provides the information about the distribution and the antibiotic resistance pattern of bacterial pathogens isolated from the UTI patients from Nepal. In addition, to our knowledge, this is the first report to indicate a possible plasmid-mediated transfer of multidrug resistance among MDR *E. coli *isolates from UTIs in Nepal. Our study showed that Enterobacteriaceae are predominant causative organisms to cause UTI, followed by Gram-positive cocci, a finding consistent with reports published elsewhere [15,16]. Among the Gram-negative bacteria, highest percents of resistance towards first-line antibiotics were found for amoxycillin (57.7%), co-trimoxazole (53.6%), and norfloxacin (36.4%). However, overall amikacin and nitrofurantoin showed a least resistance, 11.9% and 13.4%, respectively. Among Gram-positive pathogens, CoNS were found most predominant and were resistant to cephalexin (33.4%), cloxacillin (33.4%) and co-trimoxazole (33.4%) in the first-line of antibiotics. Although most of the *S. aureus *and enterococci species were found resistant to β-lactam antibiotics, fortunately resistance towards vancomycin was not observed. These results are basically in agreement with other studies [15,17].

Our report, similar to previous reports, shows that *E. coli *is the predominant causative agent of UTIs with high rate of antibiotic resistance [6,7,16,18]. Out of the 178 *E. coli *isolates, 55.6% showed resistance to amoxycillin, 54.4% to co-trimoxazole, 36.5% to norfloxacin and 35.9% to ciprofloxacin. Amoxycillin and co-trimoxazole resistance rates in our report were higher in comparison to other reports, hence empirical therapy with these antibiotics seems inadequate and should be avoided [6,18].

Among the antibiotics evaluated secondarily, amikacin was found most effective to all of the *E. coli *isolates tested. Our results show a substantial increase in the resistance to quinolones by *E. coli *isolates, an observation also reported by others [12,16,19]. Since nitrofurantoin has shown low overall *E. coli *and other isolates resistance rate in both hospitalized- and out-patients, it can be considered as the first-line therapy, a finding similar to other studies [12,20,21]. Similarly, overall resistance to aminoglycosides was comparatively low. This is consistent with previous studies of antimicrobial susceptibility in UTI infections. For examples, Aypak *et al*. showed the 6.1% resistance by *E. coli *[18], and Bean *et al*. showed the 6.7% resistance [20].

In our report, we found a high incidence of MDR UTIs in both hospitalized-patients (85%) and out-patients (36.7%), however the difference between two groups is significant. It gives the impression of extensive dissemination of MDR uropathogens in both community and hospital environment. The contributing factors for the appearance of such high bacterial antibiotic resistance in UTIs from both groups of patients may be numerous. One of them might be the injudicious use of antimicrobial agents, for example, self-medication, which is a common practice of antibiotic misuses in Nepal. In our study, we observed high incidence of UTI caused by MDR pathogens in male patients than female patients. It might be due to the reason that the incidence of UTIs among male patients is often associated with old age and/or using of mechanical devices, both of these factors have been found to contribute the multidrug resistance phenotype [22]. We observed high multidrug resistance rate in Enterobacteriaceae isolates as 7.3% *E. coli *and 18.2% *Citrobacter *spp. showed antibiotic resistance to > 5-6 classes (Table 3). In our report, the most prevalent MDR pattern shown by *E. coli *includes resistance to: amoxycillin, ciprofloxacin, cephotaxime, cefixime, co-trimoxazole, ofloxacin, norfloxacin, ceftazidime, gentamycin and ceftriaxone. Most of the MDR *E. coli *isolates showed high resistance rate to different classes of antibiotics. Our these findings are compatible with the reports from different parts of the world, including Nepal [6,16,18,21,23]. Our findings suggest that the antibiotic treatment options for UTIs caused by *E. coli *have severely impaired due to the resistance to commonly-used antibiotics, which might lead to the situation to rely only on certain antibiotics like fosfomycin, colistin and carbapenem.

We observed high prevalence of ESBL production phenotypes (55.2%) in a subset of MDR *E. coli *isolates. ESBL-producing isolates are often found to show co-resistance to many classes of antibiotics [24]. This might be a reason of high multidrug resistance phenotypes among *E.coli *isolates in our study. Especially, the increased resistance observed to fluoroquinolone, aminopenicillin and cephalosporins are striking, because these antibiotics are first choice to treat UTI in Nepal. Our data, indicating the enormous bias to bulk MDR *E. coli *isolation, hint the possibility of emergence and spread of dominant UTI- causing MDR *E. coli *strain by mutation, in the locality. Although such possibility is contradictory to other reports, where non-*E. coli *isolates also share the major part of MDR UTI isolates [6,21]. The previous reports on uropathogens and their antimicrobial susceptibility pattern from different regions of Nepal also gives the impression of existence of such situation in Nepal [25-27]. In support, a study in Denmark indicated that the mutational events, not clonal spread, are mainly responsible for the higher incidence of UTI-causing MDR *E. coli*, due to the increased use of fluoroquinolone [28]. Further molecular study including large number of isolates from different geographical regions of Nepal are needed to confirm the possibility of selection and spread of dominant MDR *E. coli *strain in the locality.

We found a wide range of plasmids of size 2 kb to 51 kb, as similar to others, in selected *E. coli *isolates. [29]. However, we did not find very large plasmids, as evidenced by other [30]. In our conjugation study, most of the transconjugants showed the MDR phenotypes and ESBL-producing phenotypes similar to donors. In our report, transconjugants having large mobilizable plasmids of size 32.5 kb, 38 kb and 51 kb were found resistant to at least amoxycillin, ciprofloxacin, cotrimoxazole, ofloxacin and norfloxacin (Table 4). Our these findings, similar to others, suggest the possibility of existence of plasmid-mediated broad-spectum multidrug resistance in uropathogens [31-34]. Additionally, we also observed the transfer of comparably small plasmids along with large plasmids by conjugation. The conjugation frequency of the MDR *E. coli *strains ranges from 0.3 × 10^-7 ^to 1.5 × 10^-7^. Similar to this, the conjugation frequency of 10^-7 ^was also found with transfer of five antibiotic resistance patterns in *E. coli *AB 1157 recipient strain [8]. Similar to donors, the MIC of the tested antibiotics towards the transconjugants was also high, except fluoroquinolones. The high MIC values shown by donors as well as recipient *E. coli *strain suggest the existence of high levels of transferable multidrug resistance in uropathogens. However, the transfer of high level fluoroquinolone resistance was not observed substantially by conjugation. This finding is consistent with other reports [35,36]. Such high levels of multidrug resistance among uropathogens have also been reported in other country [28,37]. Altogether, our findings suggest that there might be the transfer of antibiotic resistance genes by conjugation often *en bloc *in self-transferable or mobilizable plasmids, which might be responsible for the appearance of high multi drug resistance among bacterial uropathogens. Further studies involving molecular typing of antibiotic-resistant genes in plasmids are required to determine such possibility.

## Conclusion

We observed the high prevalence of multidrug resistance among bacterial uropathogens. Particularly, rate of resistance to amoxycillin, co-trimoxazole, flouroquinolones and third-generation cephalosporins were higher and these antibiotics should be avoided for empirical-based therapy of UTIs. In such cases, empirical treatment with nitrofurantoin and amikacin might provide much better antibiotic coverage. In addition, we found that the MDR *E. coli *isolates causing UTIs are capable of transferring MDR phenotypes with concomitant transfer of ESBL phenotypes to *E. coli *HB101 strain via conjugation. Altogether, our findings indicate the urgency to re-evaluate the first and second line of drugs for the antimicrobial therapy of UTIs. Regular national wise surveillance of multidrug resistance seems necessary step to combat the UTIs caused by MDR bacterial pathogens.

## Competing interests

The authors declare that they have no competing interests.

## Authors' contributions

PB has collected the sample and characterized bacterial isolates from UTI patients with the collaboration of SN. He also performed the antibiotic susceptibility testing and ESBL test with the help of SN. PB, BPM and KRG involved in the plasmid extraction and the conjugation study. BS and BL involved in study design. All authors have read the final version of manuscript and approved its finding for publication.
